# Overweight, Obesity, and Extreme Obesity Among Mississippi Adults, 2001–2010 and 2011–2015

**DOI:** 10.5888/pcd14.160554

**Published:** 2017-06-22

**Authors:** Vincent L. Mendy, Rodolfo Vargas, Gerri Cannon-Smith, Marinelle Payton

**Affiliations:** 1 Office of Health Data and Research, Mississippi State Department of Health, Jackson, Mississippi; 2 Center of Excellence in Minority Health and Health Disparities, Institute of Epidemiology and Health Services Research, School of Public Health, Jackson State University, Jackson, Mississippi

## Abstract

**Introduction:**

In 2015, about 1.5 million adults in Mississippi were overweight or obese. Obesity is associated with increased risk for diabetes and cardiovascular problems. We examined trends in the prevalence of overweight, obesity, and extreme obesity from 2001 through 2010 and 2011 through 2015.

**Methods:**

We used data from the Mississippi Behavioral Risk Factor Surveillance System to analyze trends in the prevalence of overweight, obesity, and extreme obesity among adults from 2001 through 2010 and 2011 through 2015. Joinpoint software was used to examine annual percentage change (APC) in the prevalence of each condition overall and by sex and race.

**Results:**

We observed a significant decrease in overweight prevalence from 2001 to 2010, both overall (APC, −1.3%) and among men (APC, −2.0%), blacks (APC, −1.0%), and whites (APC, −1.5%), but not among women. The overall prevalence of both obesity (APC, 2.9%) and extreme obesity (APC, 3.6%) increased significantly, and these increases occurred across all subgroups for both obesity (men APC, 3.5%; women APC, 2.5%; blacks APC, 1.9%; and whites APC, 3.8%) and extreme obesity (men APC, 6.7%; women APC, 2.5%; blacks APC, 2.2%; and whites APC, 5.0%). From 2011 to 2015, the only significant change was an increase in the prevalence of extreme obesity among whites (APC, 2.6%).

**Conclusion:**

The increasing proportion of adult Mississippians in the 2 highest-risk BMI categories warrants urgent community and clinical obesity interventions. Community-tailored and sustained obesity prevention, treatment, and control programs that include diet and physical activity are needed to address the obesity epidemic.

## Introduction

Obesity-associated health conditions include heart disease, stroke, diabetes, musculoskeletal disorders, and cancers such as endometrial, breast, and colon (1). Obesity is also associated with all-cause mortality (2,3). The high prevalence of overweight and obesity continues to pose major public health challenges both globally and in the United States, particularly in Mississippi. In 2014, more than 1.9 billion adults worldwide were overweight and more than 600 million were obese (1). In the United States, data from the 2011 through 2012 National Health and Nutrition Examination Survey indicate that 78.6 million US adults (34.9%) were obese (4). Nationally, the annual cost of medical care related to obesity is estimated at $147 billion to $210 billion (5,6). In Mississippi, about 1.5 million adults (71.3%) were overweight or obese in 2015 (7), although rates varied by race and sex (7). A study of obesity trends in the Mississippi Delta Region (a region with a disproportionately high burden of cardiovascular disease and a population that is 50% black) found that the prevalence of obesity increased significantly, 3.5% annually from 2001 to 2010 (8). The cost of obesity-related health care in Mississippi is projected to reach $3.9 billion by 2018 (9).

Given the high prevalence of obesity in Mississippi and the numerous associated health risks, information is needed on temporal trends in the prevalence of overweight, obesity, and extreme obesity in the state. Examining these trends will shed light on the burden of obesity in the state and provide information for community and population health program managers, policy makers, advocacy organizations, community faith-based organizations, and health professionals as they seek to implement obesity prevention and treatment programs and health promotion programs in the state. Such data can also be used to assess the effect of obesity programs in the state. The Mississippi State Department of Health (MSDH), in collaboration with the Centers for Disease Control and Prevention (CDC), and local stakeholders, is implementing programs — through the Mississippi Delta Health Collaborative (MDHC) — aimed at increasing access to healthy food and physical activity options in the 18-county Mississippi Delta region (www.healthyms.com/MDHC). Data on adult obesity trends, particularly the prevalence of extreme obesity in Mississippi, are limited. To fill this gap, we examined overall trends in the prevalence of overweight, obesity, and extreme obesity among Mississippi adults as well as trends by race and sex.

## Methods

### Data source

The Behavioral Risk Factor Surveillance System (BRFSS) is a state-based, random-digit–dialed telephone survey of the US noninstitutionalized civilian population aged 18 years or older. The survey is conducted in all 50 states, the District of Columbia, and 3 US territories (Puerto Rico, Guam, and the US Virgin Islands). Data from the BRFSS are reliable and valid assessments of health risk factors (10). Beginning in 2011, BRFSS data included landline and cellular telephone panels, and a new weighting method was implemented to improve accuracy (11). Survey analysts have recommended not tracking trends across 2011 because of these changes. Therefore, we analyzed trends for Mississippi respondents from 2001 through 2010 (n = 61,911) and 2011 through 2015 (n = 34,199). We reweighted the BRFSS data set by dividing the sample size of each individual year by the sum of all sample sizes corresponding to the periods 2001 through 2010 and 2011 through 2015. The BRFSS study was approved by human research review boards at departments of health in all states surveyed. Detailed information about BRFSS is available at www.cdc.gov/brfss/. Analyses were restricted to participants who self-identified as black or white; these racial groups accounted for 96.6% of the Mississippi population in the 2010 Census (8). We excluded pregnant women from the sample.

Body mass index (BMI) is a simple index of weight-for-height that is widely used to classify overweight, obesity, and extreme obesity among adults (1). BMI is defined as a person’s weight in kilograms divided by the square of their height in meters (kg/m^2^). BMI was calculated from self-reported height and weight and then categorized as follows: overweight, BMI 25.0 or greater and less than 30.0; obesity, BMI 30.0 or greater; and extreme obesity, BMI 40.0 or greater (12).

### Statistical analysis

We analyzed the data using SAS 9.4 (SAS Institute, Inc) to adjust for the disproportionate stratified sampling design of BRFSS and weighted using poststratification methods (7). Logistic regression analysis was used to test for changes over time in the prevalence of overweight, obesity, and extreme obesity. The regression models controlled for changes in the distributions by age, race, sex, annual household income, and education. The models also assessed linear and quadratic time effects by including time variables (linear and quadratic coefficients) created by coding each year with orthogonal coefficients calculated using PROC IML in SAS. Adjusted prevalence and associated standard errors were calculated by year by using SUDAAN 11 (RTI International) and were then exported to Joinpoint software (4.3.1.0) from the US Surveillance, Epidemiology, and End Results program (http://surveillance.cancer.gov/joinpoint/) to 1) determine change of direction, or joinpoint (where the prevalence trend changed direction because of a significant quadratic trend), and 2) calculate the annual percentage change (APC) for a linear or quadratic trend from 2001 to 2010 and 2011 to 2015. Analyses were constrained to minimum joinpoints (ie, 0 joinpoints, representing a straight line) when quadratic trends were not significant. Trends are described by APC (13). APCs and 95% confidence intervals (CIs) were calculated for each linear trend by fitting a regression line to the natural logarithm of the prevalence rates using calendar year as a regression variable (14). The APC would be considered significantly different from 0 at *P* values less than .05. This investigation was approved by the MSDH Institutional Review Board.

## Results

From 2001 to 2010, about half of surveyed Mississippi adults were female (51.9%), 65.4% were white, 31.6% were aged 18 to 34 years, 23.2% reported an annual household income of less than $20,000, and about half (50.9%) had more than a high school education (Table 1).

**Table 1:** Sociodemographic Characteristics of Mississippi Adults, Behavioral Risk Factor Surveillance System, 2001–2010 (n = 61,911)

Characteristic	Weighted % (95% Confidence Interval)
**Age group, y**	
18–34	31.6 (31.0–32.2)
35–49	27.4 (26.9–27.8)
50–64	23.7 (23.3–24.1)
≥65	17.3 (17.0–17.6)
**Race**	
White	65.4 (64.8–65.9)
Black	34.6 (34.1–35.2)
**Sex**	
Male	48.1 (47.5–48.6)
Female	51.9 (51.4–52.5)
**Annual household income, $**	
<20,000	23.2 (22.8–23.7)
20,000–34,999	20.8 (20.3–21.2)
35,000–49,999	13.2 (12.9–13.6)
≥50,000	28.0 (27.5–28.5)
No answer	14.7 (14.3–15.2)
**Education level**	
<High school graduate	16.5 (16.1–16.9)
High school graduate or equivalent	32.6 (32.1–33.1)
>High school graduate	50.9 (50.3–51.4)

From 2001 to 2010, overweight prevalence significantly decreased (APC, −1.3%; 95% confidence interval [CI], −1.8% to −0.9%), but there were significant increases in both obesity prevalence (APC, 2.9%; 95% CI, 2.3% to 3.5%) and extreme obesity prevalence (APC, 3.6%; 95% CI, 1.8% to 5.5%) (Table 2, Figure). Prevalence was adjusted for age, race, annual household income, and education.

**Table 2:** Adjusted Prevalence of Overweight, Obesity, and Extreme Obesity Among Mississippi Adults, 2001–2010^a^

Weight Status	Adjusted Prevalence, %^b^										Trends Determined by Joinpoint Analysis	
	2001	2002	2003	2004	2005	2006	2007	2008	2009	2010	APC^c^ (95% CI)	*P* Value
Sample size, n	3,005	4,058	4,380	5,204	4,403	6,001	7,757	7,899	11,154	8,050	NA	
**Overall**												
Overweight	37.5	36.5	36.6	36.0	36.5	34.5	35.1	33.0	33.8	33.4	−1.3 (−1.8 to −0.9)	<.001
Obesity	30.3	29.7	31.8	32.5	33.6	34.6	35.5	36.0	38.7	37.3	2.9 (2.3 to 3.5)	<.001
Extreme obesity	7.2	6.3	7.8	8.4	8.4	8.8	8.0	8.6	9.4	9.9	3.6 (1.8 to 5.5)	.002
**Sex**												
**Male**												
Overweight	46.4	43.8	43.8	43.5	42.9	40.6	41.9	38.7	38.9	38.1	−2.0 (−2.5 to −1.5)	<.001
Obesity	26.8	26.6	29.3	29.9	31.2	31.4	33.1	32.7	36.8	35.4	3.5 (2.6 to 4.4)	<.001
Extreme obesity	3.2	2.7	4.1	4.3	4.8	5.3	4.5	4.6	5.4	6.2	6.7 (3.3 to 10.3)	.002
**Female**												
Overweight	29.2	29.7	29.9	29.0	30.6	28.8	28.8	27.8	29.2	29.1	−0.4 (−1.0 to 0.3)	.22
Obesity	33.1	32.4	33.9	34.9	35.7	37.6	37.9	39.1	40.6	39.0	2.5 (1.9 to 3.2)	<.001
Extreme obesity	10.8	9.7	11.3	12.2	11.8	12.0	11.3	12.2	13.1	13.3	2.5 (1.1 to 4.0)	.004
**Race**												
**Black**												
Overweight	34.1	35.3	33.0	31.4	35.1	31.6	32.0	31.4	31.4	31.9	−1.0 (−1.9 to −0.1)	.04
Obesity	41.1	38.1	40.1	42.6	42.7	42.5	45.7	43.0	47.9	44.7	1.9 (0.8 to 3.0)	.004
Extreme obesity	10.9	9.4	11.9	13.5	13.4	11.7	11.8	12.6	13.4	13.1	2.2 (−0.2 to 4.6)	.06
**White**												
Overweight	39.3	37.2	38.5	38.4	37.3	36.1	36.7	33.9	35.1	34.1	−1.5 (−2.1 to −0.9)	.001
Obesity	24.6	25.1	27.3	27.1	28.8	30.4	30.2	32.3	33.9	33.4	3.8 (3.1 to 4.4)	<.001
Extreme obesity	5.2	4.9	5.6	5.8	5.8	7.2	6.1	6.4	7.2	8.2	5.0 (2.9 to 7.1)	<.001

Abbreviations: APC, annual percentage change; CI, confidence interval; NA, not applicable.

a Body mass index (BMI) is defined as a person’s weight in kilograms divided by the square of their height in meters. Overweight is BMI ≥25.0 and <30.0. Obesity is BMI ≥30.0. Extreme obesity is BMI ≥40.0. Pregnant women were excluded from the analysis.

b Adjusted for age, race, sex, annual household income, and education.

c APC is significantly different from 0.

**Figure:**
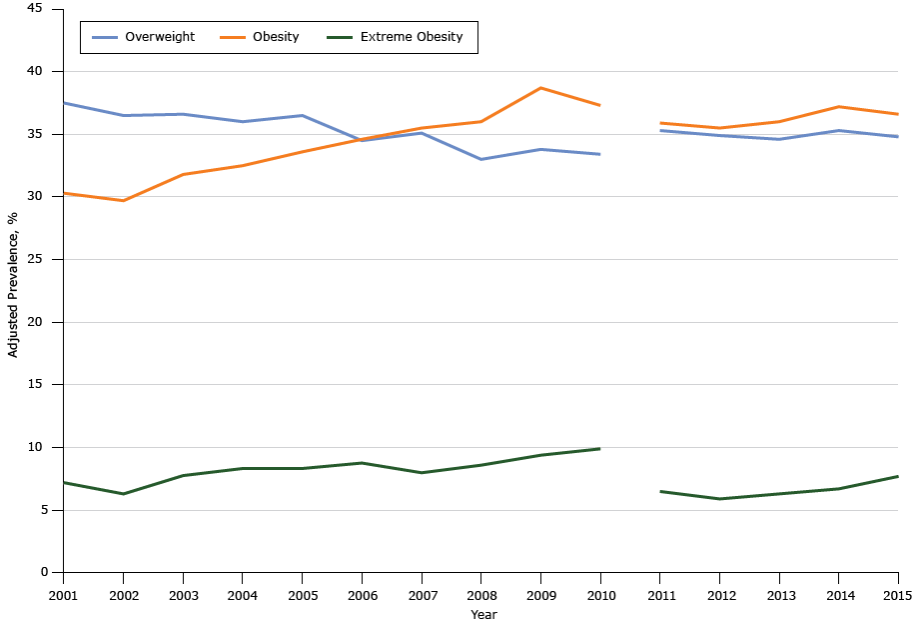
Overall trends in overweight, obesity, and extreme obesity prevalence among Mississippi adults, Behavioral Risk Factor Surveillance System, 2001–2010 and 2011–2015. Beginning in 2011, BRFSS data included landline and cellular telephone panels, and a new weighting method was implemented to improve accuracy (11). Survey analysts recommend not tracking trends across 2011 because of these changes. **Table:** 

Category	2001	2002	2003	2004	2005	2006	2007	2008	2009	2010	2011	2012	2013	2014	2015
Overweight	37.5	36.5	36.6	36.0	36.5	34.5	35.1	33.0	33.8	33.4	35.3	34.9	34.6	35.3	34.8
Obesity	30.3	29.7	31.8	32.5	33.6	34.6	35.5	36.0	38.7	37.3	35.9	35.5	36.0	37.2	36.6
Extreme obesity	7.2	6.3	7.8	8.4	8.4	8.8	8.0	8.6	9.4	9.9	6.5	5.9	6.3	6.7	7.7

This overall pattern extended to each of the 4 focal subgroups (men, women, blacks, and whites), although the magnitude of the APC varied somewhat by sex and race. From 2001 to 2010, among men, overweight prevalence significantly decreased (APC, −2.0%; 95% CI, −2.5% to −1.5%), but there were significant increases in both obesity prevalence (APC, 3.5%; 95% CI, 2.6% to 4.4%) and extreme obesity prevalence (APC, 6.7%; 95% CI, 3.3% to 10.3%). The same pattern was seen among women for a significant increase in both obesity prevalence (APC, 2.5%; 95% CI, 1.9% to 3.2%) and extreme obesity prevalence (APC, 2.5%; 95% CI, 1.1% to 4.0%); the change in overweight prevalence was not significant (APC, −0.4%; 95% CI, −1.0% to 0.3%).

From 2001 to 2010, among both blacks and whites, overweight prevalence decreased significantly (blacks: APC, −1.0%; 95% CI, −1.9% to −0.1%; whites: APC, −1.5%; 95% CI, −2.1% to −0.9%). Among both black and white respondents, there were significant increases in obesity prevalence (blacks: APC, 1.9%; 95% CI, 0.8% to 3.0%, whites, APC, 3.8%; 95% CI, 3.1% to 4.4%) and extreme obesity prevalence (blacks: APC, 2.2%; 95% CI, −0.2% to 4.6; whites: APC, 5.0%; 95% CI, 2.9% to 7.1%) (Table 2). For the subsequent 5-year period (2011–2015), the only significant change we observed was an increase in the prevalence of extreme obesity among whites (APC, 2.6; 95% CI, 1.5% to 3.8%) (Table 3). Results of the quadratic time effects were not significant and will not be further discussed.

**Table 3:** Adjusted Prevalence of Overweight, Obesity, and Extreme Obesity Among Mississippi Adults, 2011–2015^a^

Weight Status	Adjusted Prevalence, %^b^					Trends Determined by Joinpoint Analysis	
	2011	2012	2013	2014	2015	APC^c^ (95% CI)	*P* Value
Sample size, n	8,858	7,750	7,396	4,186	6,009	NA	
**Overall**							
Overweight	35.3	34.9	34.6	35.3	34.8	−0.2 (−1.2 to 0.7)	.47
Obesity	35.9	35.5	36.0	37.2	36.6	0.8 (−0.6 to 2.1)	.16
Extreme obesity	6.5	5.9	6.3	6.7	7.7	4.5 (−3.3 to 12.9)	.17
**Sex**							
**Male**							
Overweight	40.7	40.2	39.3	39.1	40.3	−0.5 (−2.2 to 1.2)	.41
Obesity	32.7	32.7	34.6	34.4	34.6	1.7 (−0.2 to 3.6)	.06
Extreme obesity	4.4	4.3	5.0	4.6	4.8	2.7 (−3.1 to 8.8)	.25
**Female**							
Overweight	30.2	29.9	30.1	31.7	29.6	0.1 (−2.5 to 2.8)	.90
Obesity	39.0	38.2	37.5	39.4	38.5	−0.1 (−2.2 to 2.0)	.88
Extreme obesity	8.4	7.4	7.6	8.6	10.5	5.9 (−5.8 to 19.0)	.22
**Race**							
**Black**							
Overweight	32.4	32.4	32.4	32.4	32.4	0.3 (−0.6 to 1.3)	.31
Obesity	44.5	43.7	43.4	43.8	44.5	−0.0 (−1.4 to 1.4)	.99
Extreme obesity	10.0	8.1	9.3	9.8	12.6	6.6 (−7.5 to 22.9)	.24
**White**							
Overweight	37.0	36.5	36.0	36.8	36.1	−0.5 (−1.5 to 0.6)	.23
Obesity	30.9	30.7	31.7	33.3	30.9	1.4 (−0.9 to 3.7)	.15
Extreme obesity	4.4	4.6	4.6	4.8	4.9	2.6 (1.5 to 3.8)	.005

Abbreviations: APC, annual percentage change; CI, confidence interval; NA, not applicable.

a Body mass index (BMI) is defined as a person’s weight in kilograms divided by the square of their height in meters. Overweight is BMI ≥25.0 and <30.0. Obesity is BMI ≥30.0. Extreme obesity is BMI ≥40.0. Pregnant women were excluded from the analysis.

b Adjusted for age, race, sex, annual household income, and education.

c APC is significantly different from 0.

## Discussion

In this study, we used the most current reliable statewide data available to examine trends in the prevalence of overweight, obesity, and extreme obesity among adult Mississippians from 2001 to 2010 and 2011 to 2015. Results were mixed, with patterns varying between the time periods. Overall, during the 10-year period from 2001 to 2010, overweight prevalence decreased significantly while obesity and extreme obesity prevalence increased significantly. The magnitude of the decline in overweight prevalence differed across subgroups during this period and was smaller among blacks than whites. The significant decrease in overweight prevalence for men, blacks, and whites is noteworthy given the perennially high prevalence of adult obesity in the state. The decrease may have occurred as the result of adults in these subgroups moving from the overweight category to either the obese or extremely obese category, because prevalence in both these categories increased. The increasing proportion of adult Mississippians in the 2 highest-risk BMI categories warrants urgent community and clinical obesity interventions in the state.

The trends in obesity for each of the 4 focal subgroups (men, women, blacks, and whites) were similar to the overall trends for 2001 to 2010. For both men and women, obesity prevalence increased significantly during this period. However, the magnitude of the increase was larger for men than for women. Similarly, the prevalence of extreme obesity increased significantly for both men and women, with the magnitude of the increase larger for men than women. For both black and white adults, obesity prevalence increased significantly from 2001 through 2010. The magnitude of the increase was larger for whites than for blacks. The prevalence of extreme obesity also increased significantly for both racial groups. For this category, the magnitude of the increase among whites was twice as large as the increase among blacks. These disparities in the magnitude of the increase in obesity prevalence need further investigation.

In the subsequent 5-year period, from 2011 through 2015, the only significant change we observed was an increase in the prevalence of extreme obesity among white adults. This finding also warrants further investigation.

Factors such as food production, food consumption, societal influences, individual psychology, individual activity, the activity environment, and biology have been found to influence obesity (15). Further research is needed to examine the influence of these factors, particularly those that relate to the environment, on obesity among Mississippi adults.

The Healthy People initiative outlines 10-year national goals for improving the lives of Americans. The Healthy People 2020 obesity objective is to reduce the US prevalence of adult obesity to 30.5%, a decrease of 10% from the 2010 prevalence (16). In 2015, more than one-third (36.0%) of adults in Mississippi were obese (7). Thus, achieving this national goal of reaching an obesity prevalence of 30.5% will require a marked reduction in adult obesity prevalence in Mississippi, which has one of the highest obesity rates in the nation. The 2015 Mississippi Obesity Action Plan goals include improving state and local support for physical activity and healthy eating across the lifespan in Mississippi; developing an intergenerational, culturally sensitive public awareness campaign on preventing obesity through healthy choices and physical activity; increasing workplace awareness of obesity; increasing the number of work sites that have environments that support wellness, including weight management, healthy food choices, physical activity, and lactation support; and increasing support in health care settings for new mothers to begin breastfeeding upon delivery and breastfeed exclusively for 6 months (17).

A statewide increase in tailored obesity-reduction initiatives, operated in partnership with local communities and advocacy organizations, can help in this endeavor. The MDHC initiative, a partnership between CDC, the MSDH, and local stakeholders, is implementing programs aimed at increasing access to healthy food and physical activity options in the 18-county Mississippi Delta region. In addition, ongoing state public health actions that work to prevent and control diabetes, heart disease, obesity and associated risk factors and to promote school health (18), operated in partnership with local stakeholders, are addressing the burden of obesity statewide.

The major strength of our study is that the results are based on data from a large sample of Mississippi adults that is representative at the state level. The study provides information that can help scholars, policy makers, community leaders, advocacy groups, public agencies (eg, parks and recreation), and health professionals address the impact of obesity in Mississippi.

The findings also have potential limitations. First, some studies have found that self-reported weight status underestimates obesity prevalence and is biased by sex (19–21); however, other studies have found self-reports to be valid and reliable (22–24). Second, the shorter length of the second time period (2011–2015) may have limited the power of the analysis to detect changes in obesity prevalence (25). Third, we could not account for others factors (eg, social norms) that affect the reporting of weight (26).

From 2001 through 2010, overweight prevalence declined significantly among Mississippi adults, while the prevalence of obesity and extreme obesity increased significantly in all subgroups. The magnitude of these changes differed across subgroups. In contrast, in the subsequent 5-year period (2011–2015), the only significant change was an increase in prevalence of extreme obesity among white adults. In Mississippi, the prevalence of obesity is lower among whites than blacks, but this finding highlights that there could be a future increase in the highest-risk BMI categories in the state as more whites move into these categories; this trend may also follow for blacks, among whom the prevalence of obesity is traditionally higher than that of whites. This could have major implications for the future obesity burden, obesity prevention, and obesity-related costs in Mississippi.

Community-tailored and sustained obesity prevention, treatment, and control programs that include diet and physical activity are needed in Mississippi to address the obesity epidemic; such efforts should first address obesity among children, young adults, and those groups that have experienced the largest increases in obesity prevalence. In addition, policies aimed at addressing the economic burden of obesity could facilitate obesity reduction in the state.
